# Whole blood transcriptome signature predicts severe forms of COVID-19: Results from the COVIDeF cohort study

**DOI:** 10.1007/s10142-024-01359-2

**Published:** 2024-05-21

**Authors:** Roberta Armignacco, Nicolas Carlier, Anne Jouinot, Maria Francesca Birtolo, Daniel de Murat, Florence Tubach, Pierre Hausfater, Tabassome Simon, Guy Gorochov, Valérie Pourcher, Alexandra Beurton, Hélène Goulet, Philippe Manivet, Jérôme Bertherat, Guillaume Assié, for the COVIDeF group

**Affiliations:** 1 Université Paris Cité, CNRS UMR8104, INSERM U1016, Institut Cochin, F-75014 Paris, France; 2 Service d’Endocrinologie, Center for Rare Adrenal Diseases, AP-HP, Hôpital Cochin, 75014 Paris, France; 3 Service de Pneumologie, AP-HP, Hôpital Cochin, 75014 Paris, France; 4 Sorbonne Université, INSERM, Institut Pierre Louis d’Epidémiologie Et de Santé Publique, AP-HP, 1901, F-75013 Paris, France; 5 Emergency Department, APHP-Sorbonne Université, Hôpital Pitié-Salpêtrière, GRC-14 BIOSFAST, CIMI, UMR 1135, Sorbonne Université, Paris, France; 6 Service de Pharmacologie, Plateforme de Recherche Clinique URC-CRC-CRB de L’Est Parisien, Assistance Publique-Hôpitaux de Paris, Hôpital Saint Antoine, Sorbonne Université, Paris, France; 7 Centre d’Immunologie Et Des Maladies Infectieuses (CIMI), Department of Immunology, Sorbonne Université, Inserm, Hôpital Pitié Salpêtrière, Groupe Hospitalo-Universitaire Assistance Publique - Hôpitaux de Paris, Paris, France; 8 Department of Infectious Diseases, Hôpital Pitié Salpêtrière, Groupe Hospitalo-Universitaire Assistance Publique - Hôpitaux de Paris, Sorbonne Université, Paris, France; 9 Service de Médecine Intensive Réanimation EOLE - Département R3S - Sorbonne, Université - Hôpital Universitaire Pitié - Salpêtrière - Assistance Publique Hôpitaux de Paris - 83 Boulevard de L’Hôpital, 75013 Paris, France; 10 UMRS 1158 Inserm-Sorbonne Université “Neurophysiologie Respiratoire Expérimentale Et Clinique’’ Intensive Care Unit, Hôpital Tenon, Assistance Publique-Hôpitaux de Paris, Paris, France; 11 Emergency Department, Hôpital Tenon, Assistance Publique-Hôpitaux de Paris, Paris, France; 12 INSERM UMR 1141 “NeuroDiderot”, Université Paris Cité, FHU I2-D2 Paris, France; 13 AP-HP, DMU BioGem, Centre de Ressources Biologiques Biobank Lariboisière/Saint Louis (BB-0033-00064), Hôpital Lariboisière, Paris, France

**Keywords:** COVID-19, Severe pneumonia, Blood transcriptome, Prediction

## Abstract

COVID-19 is associated with heterogeneous outcome. Early identification of a severe progression of the disease is essential to properly manage the patients and improve their outcome. Biomarkers reflecting an increased inflammatory response, as well as individual features including advanced age, male gender, and pre-existing comorbidities, are risk factors of severe COVID-19. Yet, these features show limited accuracy for outcome prediction. The aim was to evaluate the prognostic value of whole blood transcriptome at an early stage of the disease. Blood transcriptome of patients with mild pneumonia was profiled. Patients with subsequent severe COVID-19 were compared to those with favourable outcome, and a molecular predictor based on gene expression was built. Unsupervised classification discriminated patients who would later develop a COVID-19-related severe pneumonia. The corresponding gene expression signature reflected the immune response to the viral infection dominated by a prominent type I interferon, with *IFI27* among the most over-expressed genes. A 48-genes transcriptome signature predicting the risk of severe COVID-19 was built on a training cohort, then validated on an external independent cohort, showing an accuracy of 81% for predicting severe outcome. These results identify an early transcriptome signature of severe COVID-19 pneumonia, with a possible relevance to improve COVID-19 patient management.

**Supplementary Information:**

The online version contains supplementary material available at 10.1007/s10142-024-01359-2.

## Introduction

Since its outbreak in late 2019, the coronavirus disease 2019 (COVID-19) pandemic has seen a considerable improvement, with a decrease of detected cases and mortality worldwide (Coronavirus Disease (COVID-19) Situation Reports n. d.). However, patient management can still be challenging since the clinical course can be highly heterogeneous, with a wide spectrum of biological responses and clinical manifestations, going from mild respiratory symptoms, to lung injury and pneumonia, and, in more severe and critical cases, to multiple organ failure and death (Osuchowski et al. 2021).

From an immunological point of view, severe COVID-19 and poor prognosis associate with a specific profile showing an exaggerated immune response, characterized by a “cytokine storm”, with high levels of IL-6 and IL-10 among others, and by a dramatic changes in blood cell sub-populations, with decreased lymphocytes, T-cell subsets, eosinophils, and platelets, and increased neutrophils and neutrophils-to-lymphocytes ratio (Chen et al. 2020; Carissimo et al. 2020; Martín-Sánchez et al. 2021; Hadjadj et al. 2020; Mann, et al. 2020; Laing et al. 2020; Ahern et al. 2022). Longitudinal studies analysing the leukocyte transcriptome of COVID-19 patients have described a molecular profile characterized by robust overrepresentation of interferon-related gene expression, marked decrease of transcriptional levels of genes contributing to general protein synthesis and bioenergy metabolism, and dysregulated expression of genes associated with coagulation, platelet function, complement activation, and TNF/IL-6 signalling (Ahern et al. 2022; Gill et al. 2020; Daamen et al. 2022; Yan et al. 2021; McClain et al. 2021).

Early identification of patients at risk of progressing to severe disease is essential for patient management and for the administration of specific and individualized therapies, in order to improve patient outcome and to optimize the allocation of healthcare resources. Several laboratory biomarkers, including parameters related to increased inflammatory response (such as lymphopenia, neutrophilia, raised C-reactive protein), have been associated with COVID-19 severity, hospitalization, intensive care unit admission, and mortality (Zakeri et al. 2021). Moreover, a higher risk of severe progression and mortality is related to individual factors, including older age, male gender and the presence of diverse comorbidities, such as overweight and obesity, chronic lung disease, immune depression, diabetes, hypertension and malignancy (Li et al. 2021). Attempts to build a clinical predictor score to estimate an individual hospitalized patient’s risk of developing critical illness has been made, yet with limited accuracy (Lombardi et al. 2021; Liang et al. 2020). Up to now, it is not clear whether other markers, such as molecular classifiers built on blood-based features, can improve the early prediction of this risk. Indeed, longitudinal studies on blood transcriptome and proteome have identified strong signatures associating with COVID-19 severity (Buturovic et al. 2022; Ng, et al. 2021; Kwan et al. 2021; Lee et al. 2021). Whether these markers can be used for early prognostic prediction is not known.

Here, we performed an ancillary cross-sectional study within the prospective longitudinal COVIDeF cohort, focusing on the time of first clinical evaluation of patients with mild COVID-19 pneumonia referred to Assistance Publique-Hôpitaux de Paris hospitals (Paris, France) during the first pandemic wave. We analysed the whole blood transcriptome in order to identify an early signature predicting severe progression of the disease. A control cohort of patients with non-COVID-19 pneumonia was included to warrant the specificity of the COVID-19 prognostic signature.

## Results

### Cohort presentation

A total of 159 samples, collected from patients enrolled during the first COVID-19 outbreak (April-June 2020) and presenting mild pneumonia at the moment of first clinical evaluation, were analysed. They included 115 patients with COVID-19 diagnosis and 44 patients with pneumonia not related to COVID-19. Among the 115 patients diagnosed with COVID-19, 11 evolved towards severe pneumonia, 51 towards intermediate pneumonia and 53 towards mild pneumonia (Supplementary Table S1).

In line with established risks factors (Li et al. 2021; Bergantini et al. 2021), the risk of evolution towards severe pneumonia was associated with age, diabetes, temperature, C-reactive protein (CRP), procalcitonin, fibrinogen, neutrophils and lymphocytes at inclusion, oxygen saturation and CT scan findings (Table 1).

**Table 1:** Cohort presentation, descriptive statistics and group comparisons. *Comparison of COVID-19 patients and a group of controls presenting non-COVID-19 pneumonia, using Wilcoxon and Fisher’s tests for quantitative and qualitative variable comparisons, respectively; **Comparison of COVID-19 patients depending on their outcome (towards mild, moderate or severe pneumonia), using Kruskal–Wallis’s and Fisher’s tests for quantitative and qualitative variable comparisons, respectively. ^**§**^Neutrophils and lymphocytes scores, reflecting the scaled relative fractions inferred from transcriptome data. ^#^For hospitalisation duration, patients with fatal outcome were excluded. BMI body mass index, CRP C-reactive protein, CT scan computed tomography scan, ECMO extracorporeal membrane oxygenation, HFNO high-flow nasal oxygen, IMV Invasive Mechanical Ventilation, NIV non-invasive ventilation, PCR Polymerase Chain Reaction. Median values were reported (range values in brackets)

	Variable	Global Cohort (*N*=159)					COVID-19 Cohort (*N*=115)					
		*N*	Ctrl	COVID-19	*p*-value*	adjusted *p*-value*	N	Mild (*N*=53)	Intermediate (*N*=51)	Severe (*N*=11)	*p*-value**	adjusted *p*-value*
			(*N*=44)	patients								
				(*N*=115)								
Patients' characteristics	**Sex**	159			1.0	1.0	115				0.468	0.682
	*F*		19 (43%)	48 (42%)				21 (40%)	24 (47%)	3 (27%)		
	*M*		25 (57%)	67 (58%)				32 (60%)	27 (53%)	8 (73%)		
	**Age**	159			0.73	0.926	115				0.008	0.019
			67 (29-93)	67 (21-97)				58 (21-97)	73 (27-96)	68 (35-93)		
	**BMI**	146			0.024	0.072					0.767	0.837
			25.45 (13.9-45.9)	27.35 (13.6-42.7)				27.2 (13.6-42.2)	27.4 (14.1-42.7)	28.55 (19.1-39.5)		
Medical history	**Respiratory disease**	159			1.0	1.0	115				0.798	0.837
	*Y*		14 (32%)	36 (31%)				15 (28%)	17 (33%)	4 (36%)		
	**Cardiovascular disease**	159			0.724	0.926	115				0.078	0.151
	*Y*		22 (50%)	62 (54%)				23 (43%)	31 (61%)	8 (73%)		
	**Diabetes**	159			0.021	0.0693	115				0.022	0.048
	*Y*		3 (7%)	27 (23%)				7 (13%)	15 (29%)	5 (45%)		
	**Immunodepression**	159			0.459	0.6975	115				0.373	0.567
	*Y*		17 (39%)	37 (32%)				14 (26%)	18 (35%)	5 (45%)		
	**Corticosteroid therapy**	159			0.465	0.6975	115				0.232	0.369
	*Y*		4 (9%)	6 (5%)				1 (2%)	4 (8%)	1 (9%)		
	**Smoker status**	151			0.0001	0.00066	109				0.098	0.171
	*No*		10 (24%)	67 (61%)				30 (60%)	29 (60%)	8 (73%)		
	*Former*		19 (45%)	32 (29%)				12 (24%)	18 (38%)	2 (18%)		
	*Active*		13 (31%)	10 (9%)				8 (16%)	1 (2%)	1 (9%)		
	**Blood group**	82			0.384	0.691	58				0.614	0.749
	*A*		11 (46%)	28 (48%)				11 (42%)	15 (56%)	2 (40%)		
	*B*		1 (4%)	5 (9%)				3 (12%)	2 (7%)	0 (0%)		
	*AB*		0 (0%)	5 (9%)				1 (4%)	3 (11%)	1 (20%)		
	*0*		12 (50%)	20 (34%)				11 (42%)	7 (26%)	2 (40%)		
Blood tests at inclusion	**CRP (mg/l)**	137			0.869	1	100				1.03e-08	1.20e-07
			38.16 (1.18-340.44)	33.41 (0.54-288)				12.34 (0.54-184.7)	70.79 (3.49-288)	119.25 (4.6-143.78)		
	**Procalcitonin (µg/l)**	86			0.519	0.744	66				0.002	0.007
			0.07 (0.02-25)	0.1 (0.02-8.21)				0.06 (0.02-0.37)	0.11 (0.03-8.21)	0.18 (0.06-0.76)		
	**Fibrinogen (g/l)**	96			0.228	0.511	71				4,00E-06	2,80E-05
			4.8 (1.7-8.6)	5.46 (2.7-8.91)				4.6 (2.7-6.55)	6.27 (3.81-8.91)	5.9 (5.1-7.5)		
	**D-dimers (µg/l)**	87			0.398	0.691	65				0.491	0.687
			1579 (270-10000)	930 (270-9550)				700 (270-6170)	1280 (270-9550)	930 (290-2850)		
	**Neutrophils score**^**§**^	159			0.0013	0.00715	115				0.003	0.0095
			3.12 (0.64-6.51)	2.4 (0.47-6.42)				2.17 (0.63-4.14)	2.61 (0.47-6.42)	2.53 (1.71-5.68)		
	**Lymphocytes score**^**§**^	159			1.78e-05	0.00014685	115				0.000148	0.0007
			1.3 (0.61-2.37)	1.68 (0.49-2.43)				1.76 (0.86-2.23)	1.51 (0.49-2.43)	2.13 (1.33-2.35)		
	**Temperature (°C)**	157			0.247	0.5115	113				0.00623	0.0181
			37.25 (35.8-39.4)	37.4 (35.4-40.1)				37.15 (35.4-38.9)	37.7 (35.5-40.1)	38 (36.9-39.2)		
COVID-19 features at inclusion	**PCR +**	159			4.56e-17	1,5048E-15	115				0.078	0.151
	*Y*		0 (0%)	78 (68%)				31 (58%)	37 (73%)	10 (91%)		
	**Serology +**	159			1.09e-05	0.0001199	115				0.683	0.796
	*Y*		0 (0%)	32 (28%)				14 (26%)	16 (31%)	2 (18%)		
	**Oxygen saturation (%)**	145			0.809	0.988	104				0.00141	0.0054
			96 (70-100)	96 (74-100)				97 (87-100)	96 (83-100)	95 (74-98)		
	**Oxygen flow**	147			0.886	1	106				1.73e-12	6,055E-11
			0 (0-5)	0 (0-5)				0 (0-2)	2 (0-4)	2 (0-5)		
	**CT scan +**	145			4.25e-12	7,0125E-11	105				0.092	0.169
	*Y*		12 (30%)	94 (90%)				46 (96%)	41 (85%)	7 (78%)		
	**Pulmonary opacity**	142			0.0164	0.0671	102				0.58	0.749
	*Y*		27 (68%)	88 (86%)				41 (89%)	40 (85%)	7 (78%)		
	**Bilateral pneumonia**	145			0.0183	0.0671	105				0.575	0.749
	*Y*		7 (18%)	40 (38%)				16 (33%)	21 (44%)	3 (33%)		
	**Pulmonary embolism**	145			0.463	0.6975	105				0.814	0.837
	*Y*		4 (10%)	6 (6%)				2 (4%)	4 (8%)	0 (0%)		
	**CT scan findings**	98			0.602	0.82775	88				1.26e-07	1,1025E-06
	*Mild*		6 (60%)	35 (40%)				27 (63%)	8 (21%)	0 (0%)		
	*Moderate*		3 (30%)	38 (43%)				15 (35%)	22 (58%)	1 (14%)		
	*Severe*		1 (10%)	15 (17%)				1 (2%)	8 (21%)	6 (86%)		
COVID-19 complications	**Hospitalization**	103			-		103				0.000165	0.000721875
	*Y*		-	93 (90%)				31 (76%)	51 (100%)	11 (100%)		
	**Hospitalization duration# (days)**	93			-		93				0.000162	0.000721875
			-	8 (0-203)				4 (0-203)	10.5 (2-29)	16.5 (13-43)		
	**Ageusia**	147			0.248	0.5115	107				0.747	0.837
	*Y*		5 (12%)	23 (21%)				9 (19%)	11 (23%)	3 (27%)		
	**Anosmia**	147			0.053	0.14575	107				0.621	0.749
	*Y*		3 (8%)	23 (21%)				10 (21%)	12 (25%)	1 (9%)		
	**Thrombosis d0**	149			0.006	0.028285	107				1	1
	*Y*		6 (14%)	2 (2%)				1 (2%)	1 (2%)	0 (0%)		
	**Thrombosis any time**	159			0.318	0.617	115				0.159	0.265
	*Y*		8 (18%)	14 (12%)				4 (8%)	7 (14%)	3 (27%)		
	**NIV or HFNO**	159			1	1	115				0.00839	0.01957
	*Y*		0 (0%)	2 (2%)				0 (0%)	0 (0%)	2 (18%)		
	**IMV or ECMO**	159			1	1	115				0.00839	0.019576
	*Y*		0 (0%)	2 (2%)				0 (0%)	0 (0%)	2 (18%)		
	**Death**	159			0.191	0.484					7.53e-09	1,20E-07
	*Y*		0 (0%)	7 (6%)				0 (0%)	0 (0%)	7 (64%)		

### Unsupervised transcriptome-based classification of samples

**Fig. 1:**
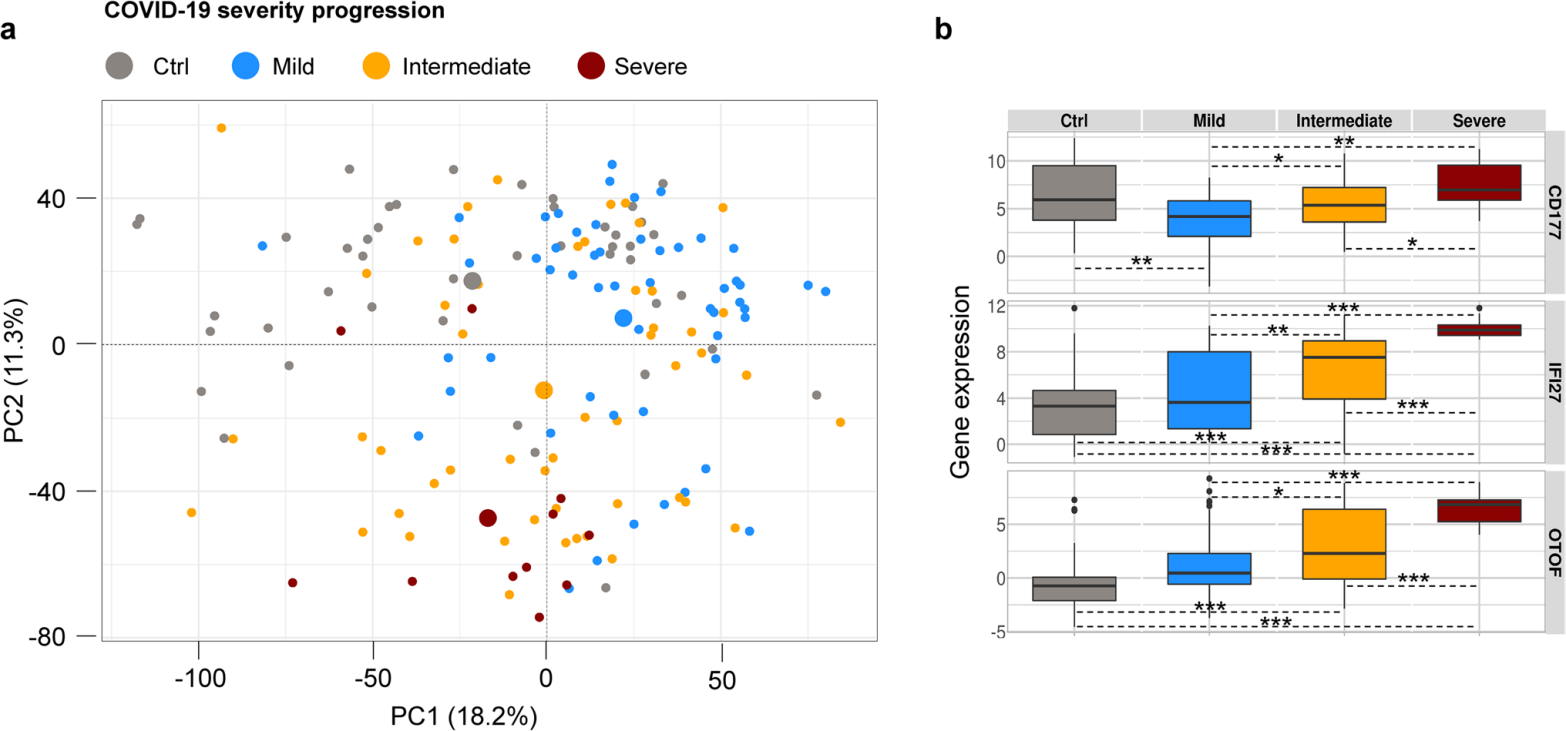
Global blood transcriptome discriminates patients depending on the type of pneumonia and the evolution of COVID-19 pneumonia. **a**) Sample projections based on the combination of the first two principal components (PC1, PC2) of unsupervised PCA performed on the whole dataset (*n* = 16001 genes, *n* = 159 samples). The center of each group is indicated by the larger circles. **b**) Boxplot of *CD177*, *IFI27* and *OTOF* gene expression in the different group of analysis. *Student’s *T*-test *p*-value < 0.05, **Student’s *T*-test *p*-value < 0.001; ***Student’s *T*-test *p*-value < 10e-6

Unsupervised principal component analysis of the whole transcriptome dataset discriminated patients with COVID-19 from controls (first principal component PC1), and patients who would later develop a severe COVID-19 pneumonia from those with mild or intermediate evolution (second principal component PC2, Fig. 1a). Over-representation analysis of the top genes most contributing to PC1 showed an enrichment in signalling pathways mainly related to neutrophil activation, while the top 100 genes most contributing to PC2 were enriched in signalling pathways related to immune response to virus infection, including complement activation, regulation of humoral immune response, response to type I interferon, and regulation of viral genome replication (Supplementary Table S2). Consistently, the most contributing gene to PC1 was *CD177*, a marker of neutrophil activation. For PC2, the most contributing genes were *IFI27*, involved in type I interferon cell response, and *OTOF*, both over-expressed in COVID-19 patients and associating with the severity of evolution (Fig. 1b).

Considering the variability possibly due to blood cell composition, we inferred the score of different blood cell subtypes for each sample (Supplementary Table S3), then we evaluated the impact of cell composition on sample classification. Globally, compared to controls, COVID-19 samples showed lower neutrophils and higher lymphocytes and lymphocyte subtypes (Table 1, Supplementary Table S4). Indeed, inverse correlation was observed between neutrophils and global lymphocytes proportion, and between neutrophils and lymphocyte T CD8 + and CD4 + memory resting subtypes in particular (Supplementary Figure S1a). However, the variability due to the global blood formula alone couldn’t properly discriminate COVID-19 patients in terms of pneumonia evolution (Supplementary Figure S1b).

### Blood early transcriptome signature of COVID-19 pneumonia

**Fig. 2:**
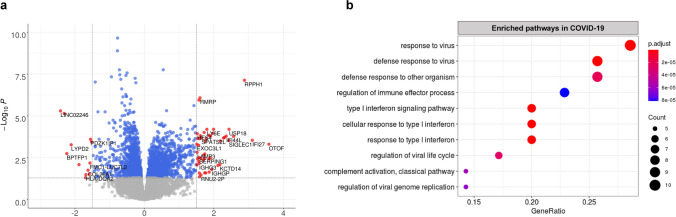
Differentially expressed genes in early COVID-19 pneumonia. **a**) Volcano plot of the top differentially expressed genes in COVID-19 (*n* = 115) versus controls (*n* = 44). b) Dot plot of the 10 most GO enriched signalling pathways of the differentially over-expressed genes in COVID-19 samples versus controls

By comparing COVID-19 samples (*n = *115) to controls (*n = *44), and after adjustment on age and blood cell composition, we identified 68 differentially expressed genes (Benjamin-Hochberg adjusted *p-*value < 0.05 and a logFC > 1.5; Supplementary Table S5), mostly over-expressed in COVID-19 (*n = *52/68). Gene ontology analysis of these over-expressed genes in the COVID-19 samples showed an enrichment in pathways related to virus response mainly involving type I interferon signalling (Fig. 2a and b; Supplementary Figure S2; Supplementary Table S6).

### Blood early transcriptome signature of future severe COVID-19 pneumonia

**Fig. 3:**
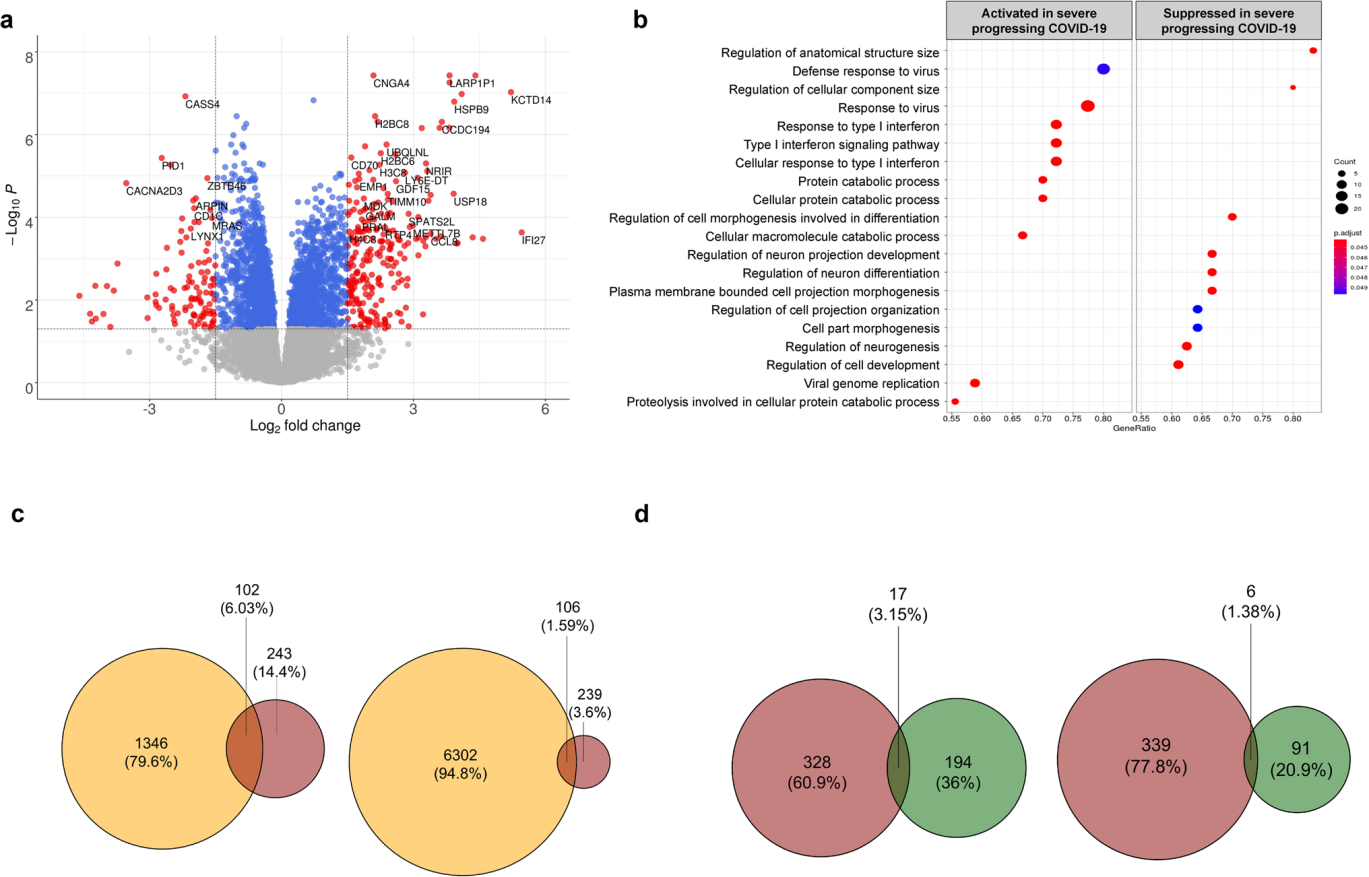
Differentially expressed genes in patients with early COVID-19 pneumonia evolving towards severity. **a**) Volcano plot of the differentially expressed genes in patients with severe (*n* = 11) versus mild (*n* = 53) evolution. **b**) Dot plot of the top 10 activated and the top 10 suppressed signalling pathways enriched for the differentially expressed genes in patients with future severe COVID-19 pneumonia. c) Venn diagram representation of differentially expressed genes in patients with severe COVID-19 pneumonia in our study (in red) and in the studies of Wang et al. and Jackson et al. (in orange) (Jackson et al. 2022; Wang et al. 2022). d) Venn diagram representation of differentially expressed genes in patients with severe COVID-19 pneumonia in our study (in red) and in patients with severe Influenza infection in the studies of Zerbib et al. and Dunning et al. (in green) (Zerbib et al. 2020; Dunning et al. 2018)

In patients with early COVID-19 pneumonia, blood samples were compared between those evolving towards severe pneumonia (*n = *11) and those remaining mild (*n = *53). After adjustment on age and blood cell composition, we identified 345 differentially expressed genes (Benjamin-Hochberg adjusted *p-*value < 0.05 and a logFC > 1.5; Supplementary Table S7). The enriched signalling pathways were represented by a response to virus infection involving a response to type I interferon, as assessed by GSEA analysis (Fig. 3a and b; Supplementary Figure S3; Supplementary Table S8).

We then tested how similar our early transcriptome signature of future severe COVID-19 pneumonia was with the longitudinal signature of evolution from mild towards severe COVID-19 pneumonia. For that aim, we analysed the overlap of our signature with published signatures reflecting the longitudinal evolution of blood transcriptome towards severe COVID-19 pneumonia (Jackson et al. 2022; Wang et al. 2022). Similarities were observed, including increased expression of *CD177*, *IFI27* and *OTOF* (Fig. 3c; Supplementary Table S9). Of note, *CD177* was also common when studying the overlap between our early signature and differentially expressed genes associated with the evolution towards severe Influenza infection (Fig. 3d; Supplementary Table S10), another virus infection, underlining the importance neutrophil induction beyond interferon activation in virus infections (Zerbib et al. 2020; Dunning et al. 2018).

### Early prediction of severe forms of COVID-19

**Fig. 4:**
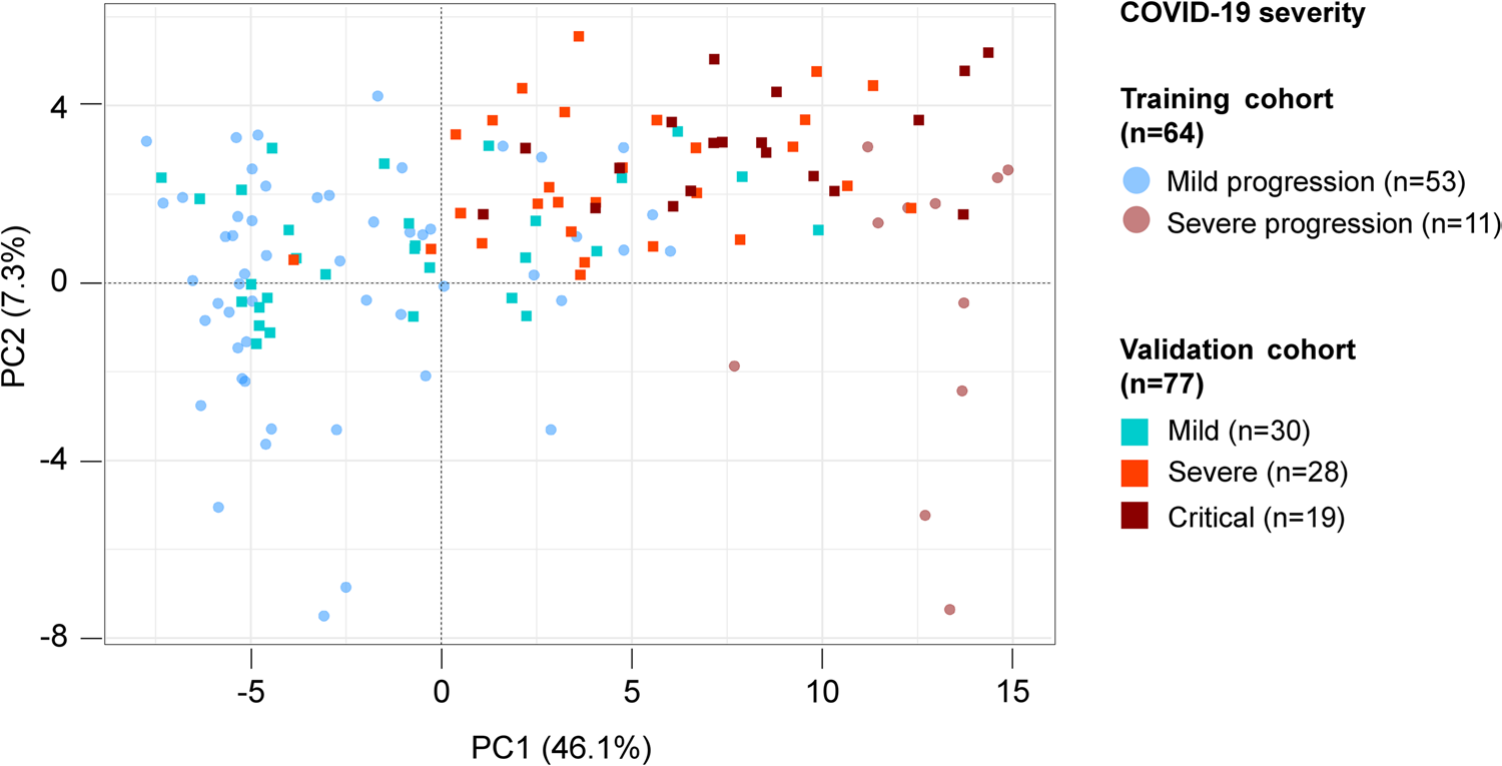
Classification of samples based on the 48 selected genes discriminating COVID-19 patients depending on pneumonia evolution. Samples projection based on the two principal components (PC1, PC2) of unsupervised PCA performed using the 48 genes selected by Elastic net regression on the training cohort. In faint circles are presented the samples from the training cohort, on which the optimization of gene selection was operated. In bright squares are presented the samples from the external independent validation cohort

To select a limited set of genes predicting severity of COVID-19, we trained an Elastic Net-penalized linear model on the sub-cohort of COVID-19 mild pneumonia patients with severe or mild evolution of the disease (*n = *11 and *n = *53, respectively), starting from the 2500 most variable genes. Forty-eight genes were selected (Supplementary Table S11), properly discriminating severe from mild evolution of COVID-19 pneumonia in the training cohort. Of note, patients with intermediate COVID-19 pneumonia outcome—not used for the training—were scattered between patients with mild and severe COVID-19 pneumonia outcome and were referred to as a grey zone. Using receiver operated characteristic (ROC) curve analysis, optimal thresholds (-0.02 and 7.69) were identified on the first component of the 48-genes principal component analysis projection (Supplementary Figures S4-6). Using an independent validation cohort of 77 patients (28 with severe outcome, 23 with intermediate outcome and 26 with mild outcome), we could confirm the classification performance (Fig. 4). Sensitivity, specificity and accuracy for predicting severe outcome were 0.64, 0.91 and 0.81 respectively (Supplementary Table S12). In a multivariate model combining the 48-genes predictor, age, sex and blood cell composition, the 48-genes predictor remained highly significant of severe outcome against mild outcome (logistic regression *p-*value < 0.001; Table 2). Of note, this signature was not discriminant between Covid and control patients (data not shown).

**Table 2:** Multivariate model combining transcriptome, age, sex, and neutrophils predictors on COVID-19 pneumonia severity. Training and validation cohorts were combined. The evolution towards severe COVID-19 pneumonia was considered. OR = Odds ratio, CI = Confidential Interval

Variables	OR	95% CI	*p*-value
48-genes predictor	1.72	1.39–2.45	< 0.001
Age	1.00	0.94–1.06	> 0.9
Neutrophils score	9.56	2.06–87.2	0.015
Sex	0.13	0.02–0.48	0.009

Post hoc analyses showed a positive correlation between the 48-genes predictor and the following biochemical variables at admission: C-reactive protein (*r* = 0.65, *p* = 2.603e-07), procalcitonin (*r* = 0.70, *p-*value = 2.073e-06) and fibrinogen (*r* = 0.62, *p-*value = 4.364e-05). Of note, diabetes, a well-established risk factor for the evolution towards severe COVID-19 pneumonia, was weakly correlated with the 48-genes predictor (*r* = 0.37, *p-*value = 0.0256).

### Ability of the 48-gene predictor of severe outcome to monitor longitudinal evolution towards severe COVID-19 pneumonia

**Fig. 5:**
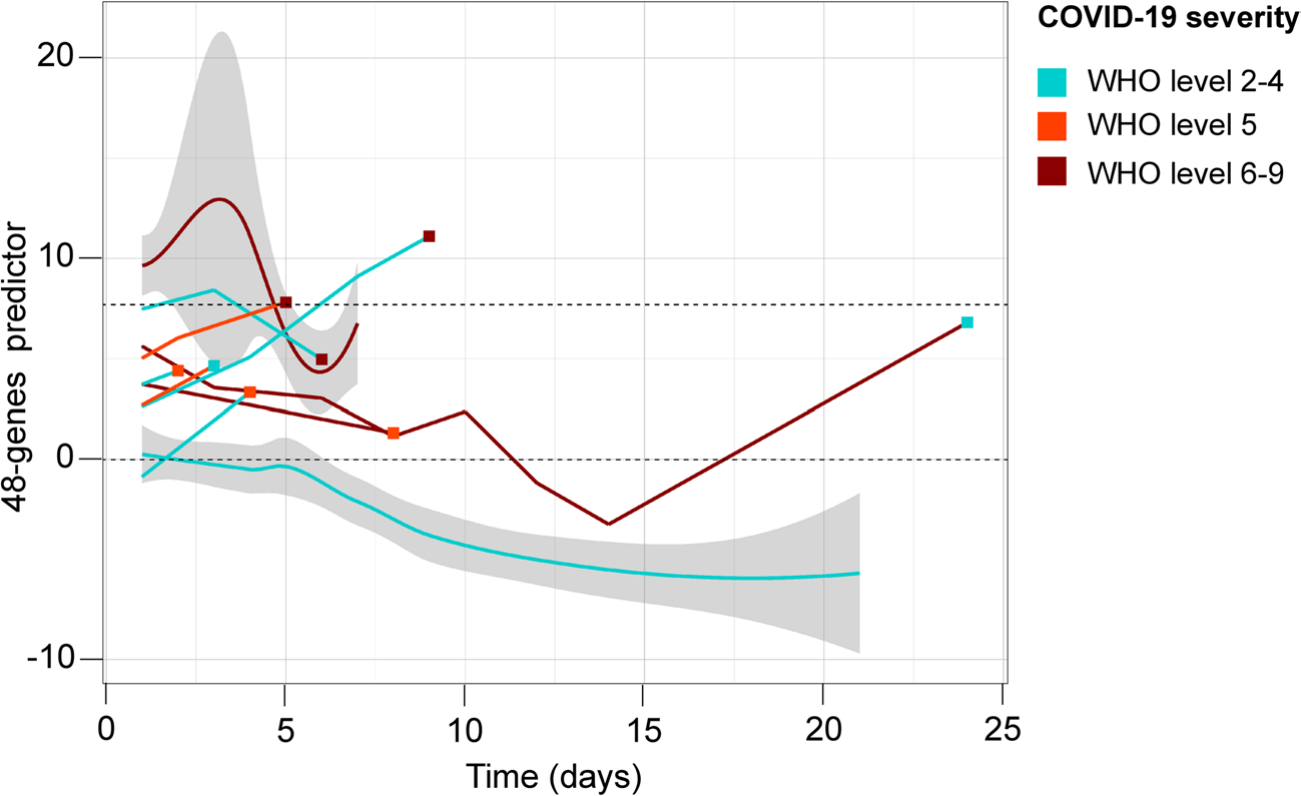
Longitudinal ability of the 48-genes predictor to monitor evolution of COVID-19 pneumonia towards severity. The red and blue curves represent the mean value of the 48-genes predictor over time in 13 patients with 6–9 and 14 patients with 2–4 WHO COVID-19 severity levels respectively. For 8 patients with a change of severity level during follow-up, individual values are provided (broken lines), with colours reflecting the severity level at each time point during follow-up. The dashed horizontal lines indicate the 48-genes predictor thresholds established on the training cohort

To explore the longitudinal performance of our transcriptomic signature, we computed our 48-gene predictor in a published cohort (Supplementary Fig. 7) (Wang et al. 2022). Globally, we found a positive correlation between the 48-genes predictor values and COVID-19 severity assessed by the WHO severity level (*r* = 0.53, *p-*value = 6.945e-16; Supplementary Fig. 8). For patients with 2 to 4 COVID-19 severity levels, the longitudinal evolution of the 48-genes predictor showed a decrease, while for patients with 6 to 9 COVID-19 severity levels, the longitudinal evolution was more variable (Fig. 5). Finally, among the 8 patients with a change in the COVID-19 severity level, with our 7.69 threshold, the 48-genes predictor was globally discriminating the patients with a worsening pneumonia (Fig. 5).

### Discussion

In this study focusing on patients with early-stage COVID-19 pneumonia, we identified a blood transcriptome signature predicting the risk of evolving towards a severe pneumonia. This signature could help to improve patients’ management, by proposing specific surveillance and treatments. This signature corresponds to a differential inflammatory profile between patients with severe and mild outcome, with the implication of humoral immune response, complement activation and interferon signalling pathway. This signature includes the over expression of inflammation markers previously reported in patients with severe COVID-19 pneumonia, such as *IFI27* (Shojaei et al. 2023) or *CD177* (Jackson et al. 2022; Wang et al. 2022; Lévy et al. 2019; An et al. 2021; An et al. 2022).This signature shows a gradient of expression from mild to intermediate and severe forms. Thus, the inflammatory signature observed in patients with severe COVID-19 pneumonia seems to be present early in the course of the disease, and to reflect the risk of developing a severe outcome.

Based on this observation we designed a predictor, with optimal selection of transcriptome biomarkers able to classify patients depending on their COVID-19 pneumonia evolution. This predictor could be validated on an independent validation cohort, where patients were evaluated longitudinally. However, with an accuracy of 0.81 the prediction of evolution towards a severe COVID-19 pneumonia is not absolute. This relates to the limited sensitivity in the validation cohort, with some COVID-19 patients with severe outcome grouped with patients with intermediate outcome. Nevertheless, taking into account the importance of both identifying early patients at risk of evolving towards a severe pneumonia and optimizing healthcare resources, in the validation cohort, none of the patients with severe COVID-19 pneumonia outcome were predicted as mild and only one patient with an actual mild COVID-19 pneumonia outcome was predicted as severe. In addition, the clinical criteria for classifying COVID-19 patients in the validation cohort, and the time of sampling during the course of the disease may have impacted the evaluation of accuracy on the validation cohort. Another limitation of the prediction of outcome is the broad distribution of patients evolving towards intermediate forms of pneumonia, with some overlap with patients evolving towards mild pneumonia -mainly in the training cohort-, and with those evolving towards severe pneumonia -mainly in the validation cohort. Though being important, the proper evaluation of intermediate patients is difficult for two reasons. Firstly, no clear clinical definition is available, with variable diagnostic criteria and thresholds to define intermediate Covid pneumonia (Jackson et al. 2022; Wang et al. 2022). Including these intermediate patients would have increased the size of the training cohort, but also the risk of misclassification. Limiting the inclusion to “mild” and “severe” classes warranted well-defined labels for training the predictor. Secondly, the cohort size is not large enough to assess the existence of statistically significant thresholds in the 48-genes predictor in patients with intermediate COVID-19 pneumonia. However, in our study, these intermediate patients indisputably fall in-between patients with mild and severe outcome as a continuum and they probably represent patients who deserve a closely clinical surveillance thus globally strengthening the validity of transcriptome prediction. This grey zone may also reflect a biological variability, and thus a certain limitation of the ability to predict outcome with this technique. Another potential issue in this study is the risk of overfitting due to the limited number of patients and the high number of features. This risk was mitigated by the cross-validation strategy in the training cohort, and the validation in two independent cohorts. Finally, a post hoc association between the 48-genes predictor and clinical and biochemical variables showed several significant associations. However, the prognostic value of these associated variables could not be tested in the final multivariate model due to the limited cohort size, and to the limited data available in the independent validation cohort.

This prognostic signature appears specific to COVID-19 patients, compared to controls (patients with non-COVID-19 pneumonia). Compared to controls, COVID-19 patients present a transcriptome signature reflecting pathways related to virus response mainly involving type I interferon response. Type 1 interferon activation has been well established when COVID-19 progresses towards severe pneumonia in longitudinal series, with several markers reported including IFI27, SIGLEC1, OAS1/2, IFI44, IFI44L, ISG15 (Shaath et al. 2020; Krämer et al. 2021; Masood et al. 2021; Khorramdelazad et al. 2022; Xu et al. 2022). Another potentially relevant gene identified here is *OTOF*, associated with inflammation and described as a type I IFN-induced effector (Roberson et al. 2022; Ding et al. 2022). Our results show that interferon type 1 activation occurs early in the course of COVID-19 pneumonia. This signature could contribute to diagnose the SARS-CoV-2 infection.

In addition, beyond COVID-19 pneumonia, to which extent this type 1 interferon signature is systematically present in SARS-CoV-2 infected patients remains to be established, especially in patients with mild or asymptomatic forms of the disease.

This study comes after several publications showing the implication of type I interferon in the evolution towards severe COVID-19 pneumonia. However, contrasting with a vast majority of previous works, this study is focusing on early-stage patients, when COVID-19 pneumonia is still mild. The clinical characterization is quite extensive, and the follow-up well documented, enabling a proper classification in terms of outcome. This original design was required for demonstrating the existence of a signature predicting the outcome. Of note, the inclusion of patients is restricted to the first outbreak wave in the training cohort. To which extent do these signatures stand with the new SARS-COV2 variants remains to be established. In addition, the relative proportion of severe pneumonia in the subsequent outbreak waves decreased, in the context of the progressive immunisation of population through vaccines and history of COVID-19 infections. However, severe pneumonia still occurs, and disease complications are still challenging to predict at individual levels. The early transcriptome signature proposed here may help improving this challenging detection.

In conclusion, whole blood transcriptome is able to early predict the outcome of COVID-19 pneumonia. This discrimination mainly relies on type 1 interferon activation, along with other immune alterations, which are already present at an early stage of the disease in patients later developing a severe pneumonia.

## Methods

### Patients and samples

A total of 159 patients presenting an early-stage pneumonia at the moment of first clinical evaluation at the hospital were recruited prospectively between April and June 2020 in Assistance Publique-Hôpitaux de Paris hospitals (Paris, France), as part of the multicentre longitudinal COVIDeF cohort (NCT04352348). Early-stage COVID-19 pneumonia was defined as confirmed SARS-Cov-2 infection, requiring at the admission supplemental oxygen but not ≥ 6 L/minute, or characterized by oxygen saturation < 95% or by the presence of one or more pneumonia morphologic criteria (CT scan or chest X-ray). COVID-19 diagnosis was made for 115 patients, based on positive SARS-CoV-2 PCR and/or serology test (*n = *79), and/or on the presence of typical clinical symptoms with CT findings (*n = *36). Whole blood samples for transcriptome analysis were collected at the moment of first clinical evaluation and only patients with an early-stage pneumonia were included. Disease evolution was evaluated on a time lap of 14 days after inclusion, and patients’ status was classified as mild (*n = *53), intermediate (*n = *51) or severe (*n = *11) pneumonia, according to the onset and evolving severity of COVID-19 complications, including hospitalization duration, need for oxygen supply, mechanical ventilation, or extra corporeal oxygenation, death. Specifically: i) patients who didn’t need oxygen supply, either hospitalized or not, were classified as having mild progression COVID-19 pneumonia; ii) patients who were hospitalized with standard oxygen supply were classified as having intermediate progression COVID-19 pneumonia; iii) patients who were hospitalized and received mechanical ventilation, or extra corporeal oxygenation, or deceased, were classified as having severe progression COVID-19 pneumonia. Cases of pneumonia of other etiology (*n = *44), used as controls, corresponded to patients with negative PCR and not diagnosed as COVID-19 by the physician based on CT findings.

### RNA collection and extraction

Whole blood samples were collected into PAXgene tubes (PreAnalytiX, Hombrechtikon, Switzerland), following the manufacturer’s instruction. Total RNA was extracted on a QIAcube extractor, following the manufacturer’s instruction (Qiagen, Hilden, Germany), at the CRB platform (Saint Antoine hospital, Paris). Quantification and quality control of RNA were performed on a 2100 Bioanalyser System (Agilent Technologies, Inc., Santa Clara, CA, US). All samples passed the integrity quality control (RIN > 7).

### Transcriptome data generation

Quantification and quality control of nucleic acids was performed by capillary migration on a Fragment Analyzer (Agilent Technologies, Inc.). Starting from 100 ng total RNA, mRNAs poly(A) were selected using oligo dT magnetic beads (NEBNext® Poly(A) mRNA Magnetic Isolation Module, New England Biolabs, Ipswich, MA, US), fragmented at around 300 bp and converted to oriented DNA (NEBNext® Ultra™ II RNA First Strand Synthesis Module & Directional RNA Second Strand Synthesis Module, New England Biolabs). Size selection and purification were performed using magnetic beads (Sera-Mag magnetic beads, GE Healthcare, Chicago, IL, US), and libraries were prepared (NEBNext® Ultra™ II End repair/A-tailing Module & Ligation Module, New England Biolabs), amplified by PCR (KAPA Hifi HotStart ReadyMix, Roche, Basil, Switzerland), quantified by qPCR (NEBNext® Custom 2X Library Quant Kit Master Mix, New England Biolabs; QuantStudio 6 Flex Real-Time PCR System, Life Technologies, Carlsbad, CA, US) and the related size profile was analyzed by capillary migration on a Fragment Analyzer (Agilent Technologies, Inc.). *Paired-end* sequencing (twice 100 cycles) was performed by “sequencing-by-synthesis” technology on a Flow Cell S2 NovaSeq 6000 platform (Illumina, San Diego, CA, US). Transcript quantification was done using the Salmon tool (Patro et al. 2017) (v.1.4.0) on transcriptome reference from GENCODE (Frankish et al. 2021) (release 33—GRCh38.p13).

### Bioinformatics analyses

Quality control was performed on raw count matrix. All samples passed this control. Counts were aggregated for transcripts corresponding to the same gene, and only genes with a count sum > 0 in all samples were further considered. Globin genes were also discarded, as previously published (Harrington et al. 2020).

Counts were normalized with *DESeq2* (Love et al. 2014) (v.1.24.0). From gene counts, blood cell composition was inferred using the online CIBERSORTx tool (Stanford University 2022) (Newman et al. 2019), with the following parameters: B-mode batch correction, disabled quantile normalization, absolute mode, *n = *500 permutations. For each cell types, a score is generated, that reflects the abundance of each cell type in a mixture. Given the high correlation of each blood cell type proportion with neutrophils proportion (Supplementary Figure S1a), neutrophils proportion was chosen as a unique proxy of cell blood composition.

The *edgeR* package (Robinson et al. 2010) (v.3.26.8) was used to read and pre-process the data before analysis: raw counts were converted to counts per million (CPM), and lowly expressed genes were removed using a CPM > 1 in at least 3 samples as cut-off, obtaining a final dataset of *n = *16,001 genes and *n = *159 samples. Normalization was then performed by using the trimmed mean of M-values (TMM) method (Robinson and Oshlack 2010), as implemented in the *edgeR* package. The same packages and method were used to process and normalize data from the validation cohort (Ahern et al. 2022) and the additional longitudinal cohort (Wang et al. 2022). Global data structure was assessed on log2-CPM of normalized data by unsupervised principal component analysis (PCA). This method was chosen for the interpretability of PCA axes for deciphering the biological meaning of the observed variability. Over-representation analysis of genes most contributing to PCA components was performed by using the *clusterProfiler* package (Wu et al. 2021) (v.3.12.0). Of note, the *edgeR* normalisation did not significantly modify the normalized expression levels compared to CIBERSORTx (gene expression correlation *r* = 0.999, *p-*value < 2.2e-16).

To remove heteroscedasticity of counts data, normalized data were transformed using the *voom* function (Liu et al. 2015) implemented in the *edgeR* package. Differential expression analysis was performed by applying linear modelling using the *limma* package (Ritchie et al. 2015) (v. 3.40.6), including the estimated neutrophils count and age as covariates in the model matrix. Differentially expressed genes were selected using a Benjamin-Hochberg adjusted *p-*value < 0.05 and a logFC > 1.5 as cut-offs. Gene set enrichment analysis (GSEA) of differentially expressed genes was performed using the *clusterProfiler* package.

For predicting COVID-19 severity from transcriptome, gene selection was performed on the sub-cohort of severe and mild cases, by fitting an Elastic Net regularized regression (α = 0.5) on the most variable genes (*n = *2500), with a tenfold cross-validation, using the *glmnet* package (Friedman et al. 2010) (v. 4.1–1). The predictive model, combining 48 discriminating genes, was assessed on an independent validation cohort from the whole blood RNAseq dataset recently published by *Ahern et al*^*9*^. Seventy-seven samples were selected (26 mild, 23 intermediates and 28 severe COVID-19 pneumonia, based on classification criteria similar to those we used in our cohort), using the following criteria: confirmed COVID-19 diagnosis and last sample for the same patient with multiple sampling time points.

Another cohort was used to explore the ability of the 48-genes predictor to monitor the longitudinal evolution of patients (Wang et al. 2022). PCA using the 48-genes predictor was performed using the PCA weights from the training cohort.

## Statistical analyses

Quantitative variable correlations were performed using Pearson’s test. Quantitative and qualitative variable comparisons between groups were performed using Kruskal–Wallis’s test and Fisher’s test, respectively. ROC curve analysis was performed and the optimal thresholds predictive of COVID-19 outcome were defined using Youden’s J index. A multivariate analysis was performed on the combined samples of the training and the external independent cohorts, using a logistic regression model on 4 variables: the 48-genes predictor (based on the PC1 coordinates of the 48-genes principal component analysis projection), the neutrophils score, and the patients’ age and sex. All *p-*values were two-sided and adjusted for multiple comparisons using Benjamini-Hochberg’ method. The level of significance was set at adjusted *p-*value < 0.05. All tests were computed in R software environment.

### Supplementary Information

Below is the link to the electronic supplementary material.

Supplementary file1 (Supplementary Figure S1) (TIFF 3068 KB) Blood cell composition inferred from transcriptome. a) Correlation plot between the inferred proportion of different blood cell subtypes. b) Blood cell composition on its own poorly discriminates patients depending on COVID-19 pneumonia evolution. Sample projections based on the combination of the first two principal components (PC1, PC2) of unsupervised PCA performed on the inferred proportion of blood cell subtypes (i=20 blood cell types, *n*=159 samples). The center of each group is indicated by the larger circles

Supplementary file2 (Supplementary Figure S2) (TIFF 3012 KB) Whole blood early transcriptome signature of COVID-19 pneumonia. Unsupervised clustering of samples using the 68 differentially expressed genes in the comparison COVID-19 pneumonia versus controls, after adjustment on age and blood cell composition

Supplementary file3 (Supplementary Figure S3) (TIFF 2586 KB) Whole blood early transcriptome signature of patients evolving towards severe COVID-19 pneumonia. Unsupervised clustering of samples using the 100 most significant differentially expressed genes in the comparison of patients with evolution towards severe COVID-19 pneumonia versus those evolving towards mild COVID-19 pneumonia, after adjustment on age and blood cell composition

Supplementary file4 (Supplementary Figure S4) (TIFF 82 KB) ROC curve analysis and optimal thresholds for COVID-19 pneumonia outcome discrimination between patients with mild and intermediate outcome on the training cohort. The optimal threshold is -0.02 (AUC = 0.80)

Supplementary file5 (Supplementary Figure S5) (TIFF 80 KB) ROC curve analysis and optimal thresholds for COVID-19 pneumonia outcome discrimination between patients with intermediate and severe outcome on the training cohort. The optimal threshold is 7.69 (AUC = 0.98)

Supplementary file6 (Supplementary figure S6) (TIFF 196 KB) Discrimination of samples based on the 48 selected genes discriminating COVID-19 pneumonia evolution. Samples projection based on the two principal components (PC1, PC2) of unsupervised PCA performed using the 48 genes selected by Elastic net regression on the training cohort. In faint circles are presented the COVID-19 pneumonia samples with mild and severe evolution from the training cohort (*n*=64), on which the optimization of gene selection was operated. In bright squares are presented the COVID-19 pneumonia samples with intermediate evolution. The dashed red lines indicate optimal thresholds for COVID-19 pneumonia outcome discrimination (determined on the training cohort)

Supplementary file7 (Supplementary Figure S7) (TIFF 1189 KB) PCA projection of 203 additional samples (bright squares), based on the 48-genes selection using the PCA weights established on the training cohort (faint circles)

Supplementary file8 (Supplementary Figure S8) (TIFF 867 KB) Correlation analysis between the 48-genes predictor and COVID-19 WHO severity levels in the Wang *et al* cohort^24^

Supplementary file9 (XLSX 28 KB) - Supplementary Table S1: Patients diagnostic group.

Supplementary file10 (XLSX 18 KB) - Supplementary Table S2: Gene ontology enrichment on the first two axes of the principal component analysis of patients’ transcriptome, including COVID-19 and non-COVID-19 pneumonia.

Supplementary file11 (XLSX 45 KB) - Supplementary Table S3: Blood cell composition at inclusion, inferred from transcriptome data.

Supplementary file12 (XLSX 11 KB) - Supplementary Table S4: Blood cell composition at inclusion: comparison between the different groups of pneumonia.

Supplementary file13 (XLSX 16 KB) - Supplementary Table S5: Differentially expressed genes in COVID-19 pneumonia versus controls (Limma analysis).

Supplementary file14 (XLSX 14 KB) - Supplementary Table S6: Gene ontology enrichment of the over-expressed genes in the COVID-19 samples.

Supplementary file15 (XLSX 36 KB) - Supplementary Table S7: Differentially expressed genes in patients with severe COVID-19 pneumonia outcome versus patients with mild COVID-19 pneumonia outcome (Limma analysis).

Supplementary file16 (XLSX 14 KB) - Supplementary Table S8: Gene set enrichment analysis of the over-expressed genes in severe COVID-19 pneumonia samples.

Supplementary file17 (XLSX 11 KB) - Supplementary Table S9: Intersection of DEGs in severe COVID-19 found in our study and in the studies of Wang et al24 and Jackson et al23.

Supplementary file18 (XLSX 10 KB) - Supplementary Table S10: Intersection of DEGs in severe COVID-19 found in our study and in severe Influenza infection in the studies of Zerbib et al25 and Dunning et al26.

Supplementary file19 (XLSX 15 KB) - Supplementary Table S11: Forty-eight genes properly discriminating severe from mild evolution of COVID-19 pneumonia in the validation cohort (Elastic Net-penalized linear model).

Supplementary file20 (XLSX 10 KB) - Supplementary Table S12: Confusion matrix for the COVID-19 pneumonia outcome prediction on the validation cohort.

## Data Availability

The dataset generated and analysed during the current study is available in the EMBL-EBI BioStudies repository (reference number: S-BSST1135; https://www.ebi.ac.uk/biostudies/studies/S-BSST1135?key=62d4dc30-e0d4-4f4b-891f-58c5777a0cd3).
