# Supplementary figures and images for: Whole blood transcriptome signature predicts severe forms of COVID-19: Results from the COVIDeF cohort study
Source: Funct Integr Genomics. 2024 May 21;24(3):107. doi: 10.1007/s10142-024-01359-2 (PMC11108918; doi:10.1007/s10142-024-01359-2)

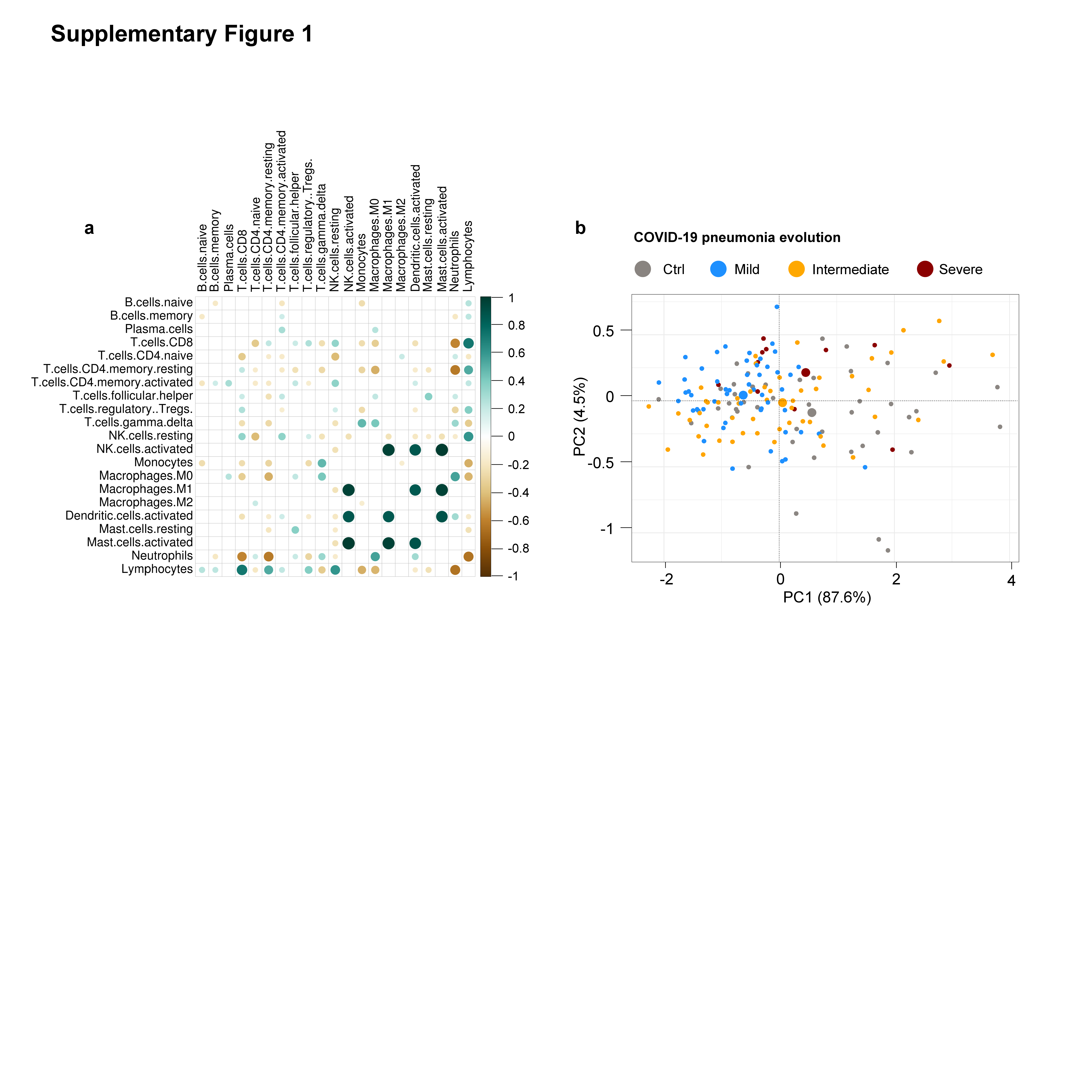

Supplement: Supplementary file 1 — Supplementary file1 (Supplementary Figure S1) (TIFF 3068 KB) Blood cell composition inferred from transcriptome. a) Correlation plot between the inferred proportion of different blood cell subtypes. b) Blood cell composition on its own poorly discriminates patients depending on COVID-19 pneumonia evolution. Sample projections based on the combination of the first two principal components (PC1, PC2) of unsupervised PCA performed on the inferred proportion of blood cell subtypes (i=20 blood cell types, n=159 samples). The center of each group is indicated by the larger circles [file 10142_2024_1359_MOESM1_ESM.tiff]

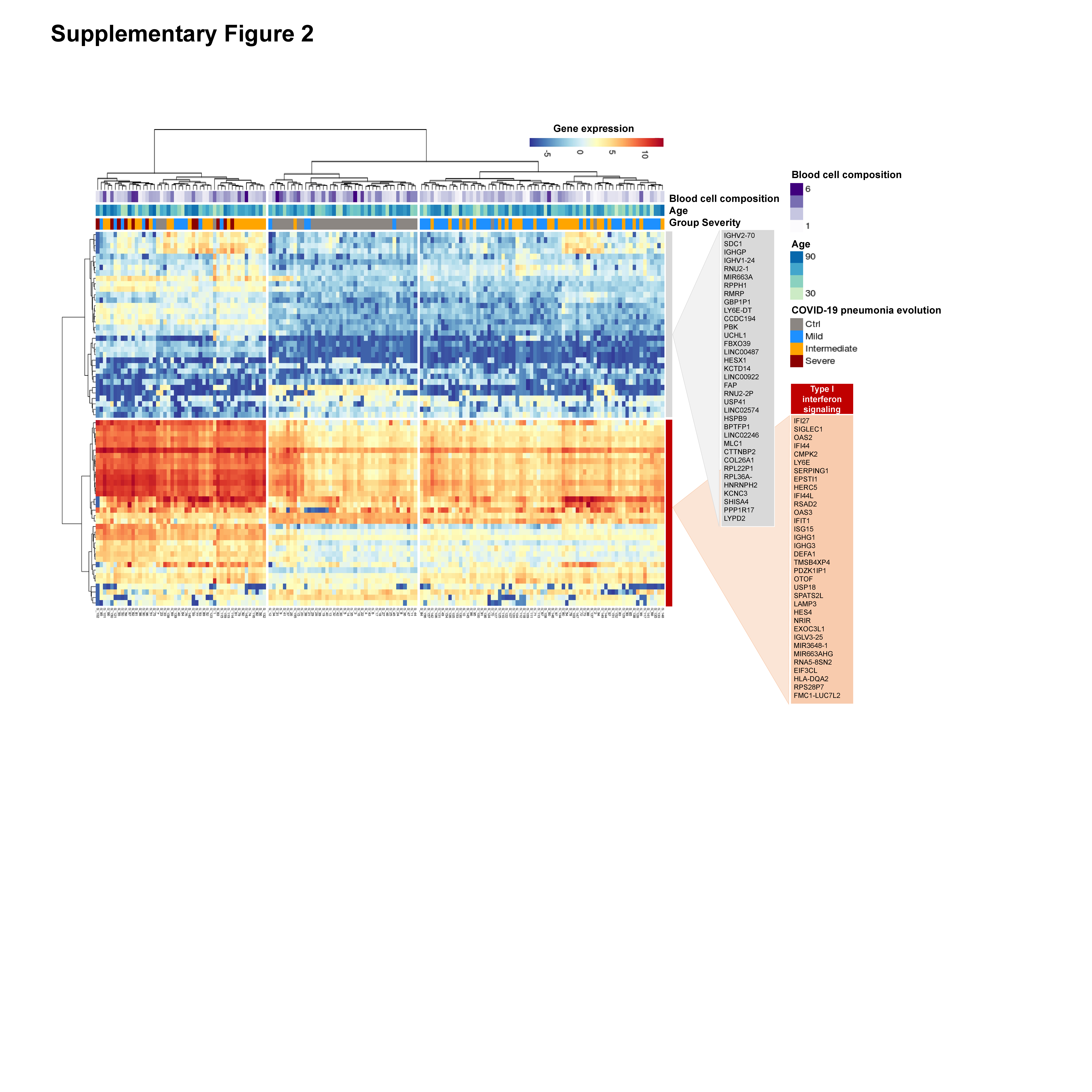

Supplement: Supplementary file 2 — Supplementary file2 (Supplementary Figure S2) (TIFF 3012 KB) Whole blood early transcriptome signature of COVID-19 pneumonia. Unsupervised clustering of samples using the 68 differentially expressed genes in the comparison COVID-19 pneumonia versus controls, after adjustment on age and blood cell composition [file 10142_2024_1359_MOESM2_ESM.tiff]

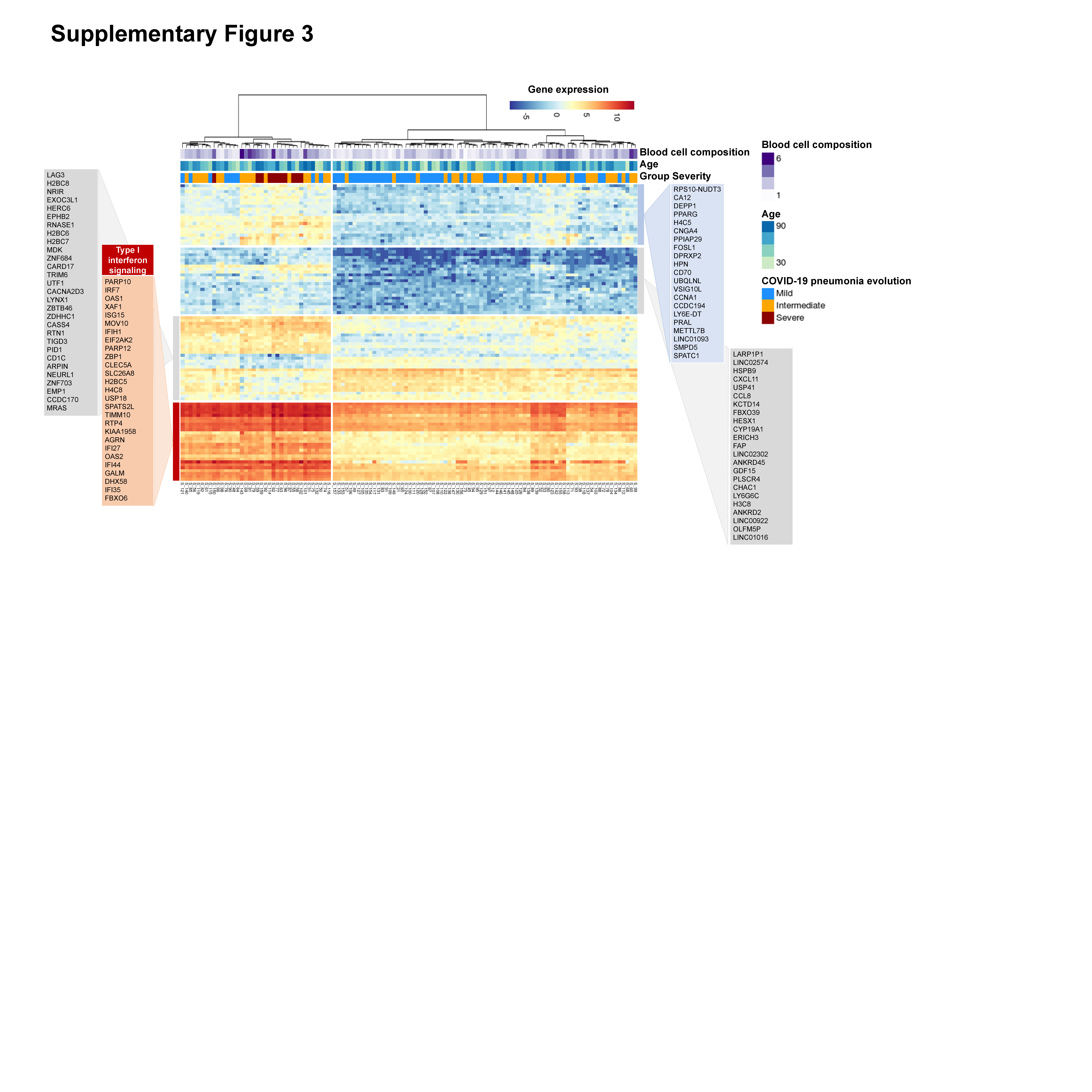

Supplement: Supplementary file 3 — Supplementary file3 (Supplementary Figure S3) (TIFF 2586 KB) Whole blood early transcriptome signature of patients evolving towards severe COVID-19 pneumonia. Unsupervised clustering of samples using the 100 most significant differentially expressed genes in the comparison of patients with evolution towards severe COVID-19 pneumonia versus those evolving towards mild COVID-19 pneumonia, after adjustment on age and blood cell composition [file 10142_2024_1359_MOESM3_ESM.tiff]

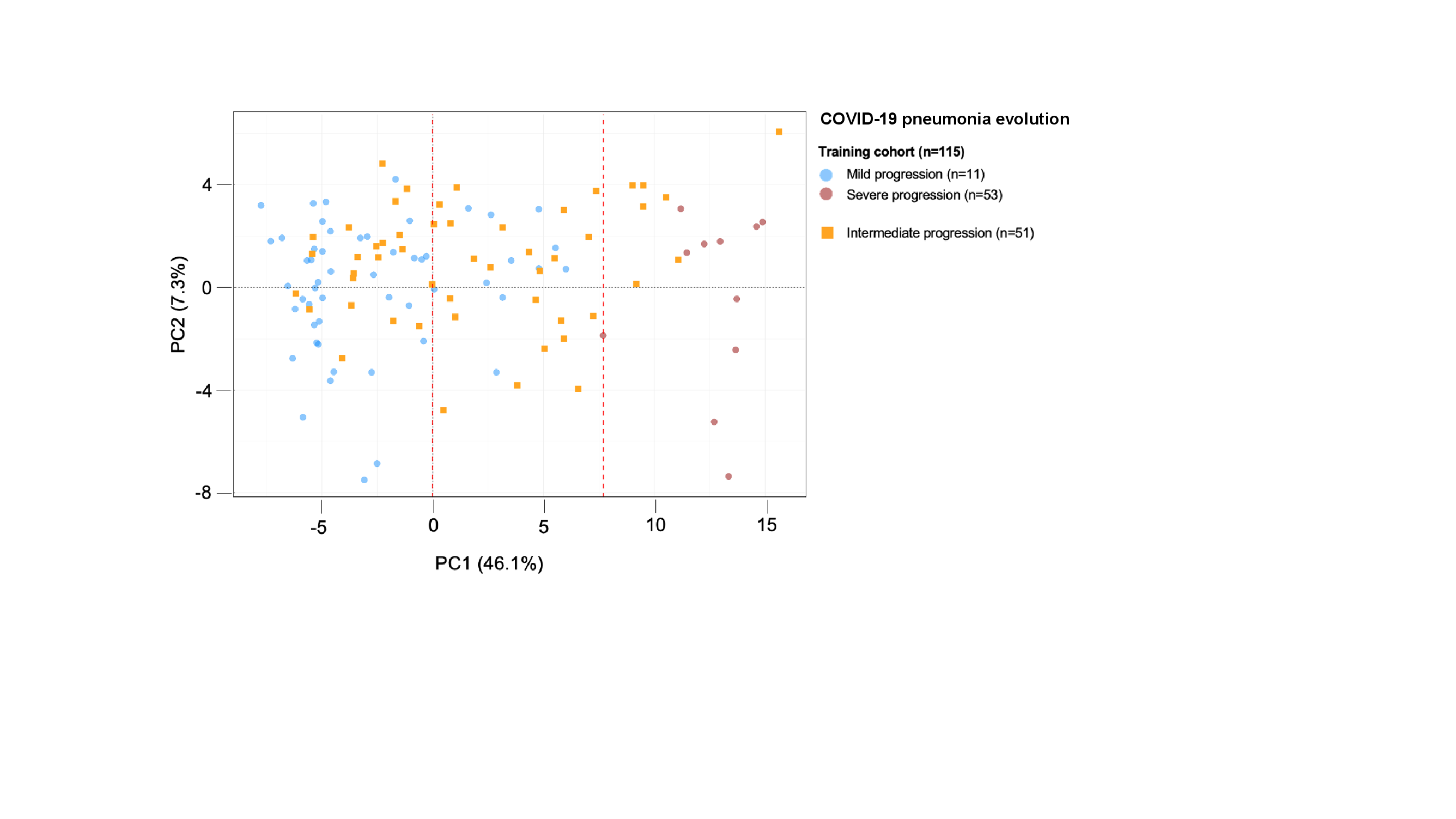

Supplement: Supplementary file 6 — Supplementary file6 (Supplementary figure S6) (TIFF 196 KB) Discrimination of samples based on the 48 selected genes discriminating COVID-19 pneumonia evolution. Samples projection based on the two principal components (PC1, PC2) of unsupervised PCA performed using the 48 genes selected by Elastic net regression on the training cohort. In faint circles are presented the COVID-19 pneumonia samples with mild and severe evolution from the training cohort (n=64), on which the optimization of gene selection was operated. In bright squares are presented the COVID-19 pneumonia samples with intermediate evolution. The dashed red lines indicate optimal thresholds for COVID-19 pneumonia outcome discrimination (determined on the training cohort) [file 10142_2024_1359_MOESM6_ESM.tiff]

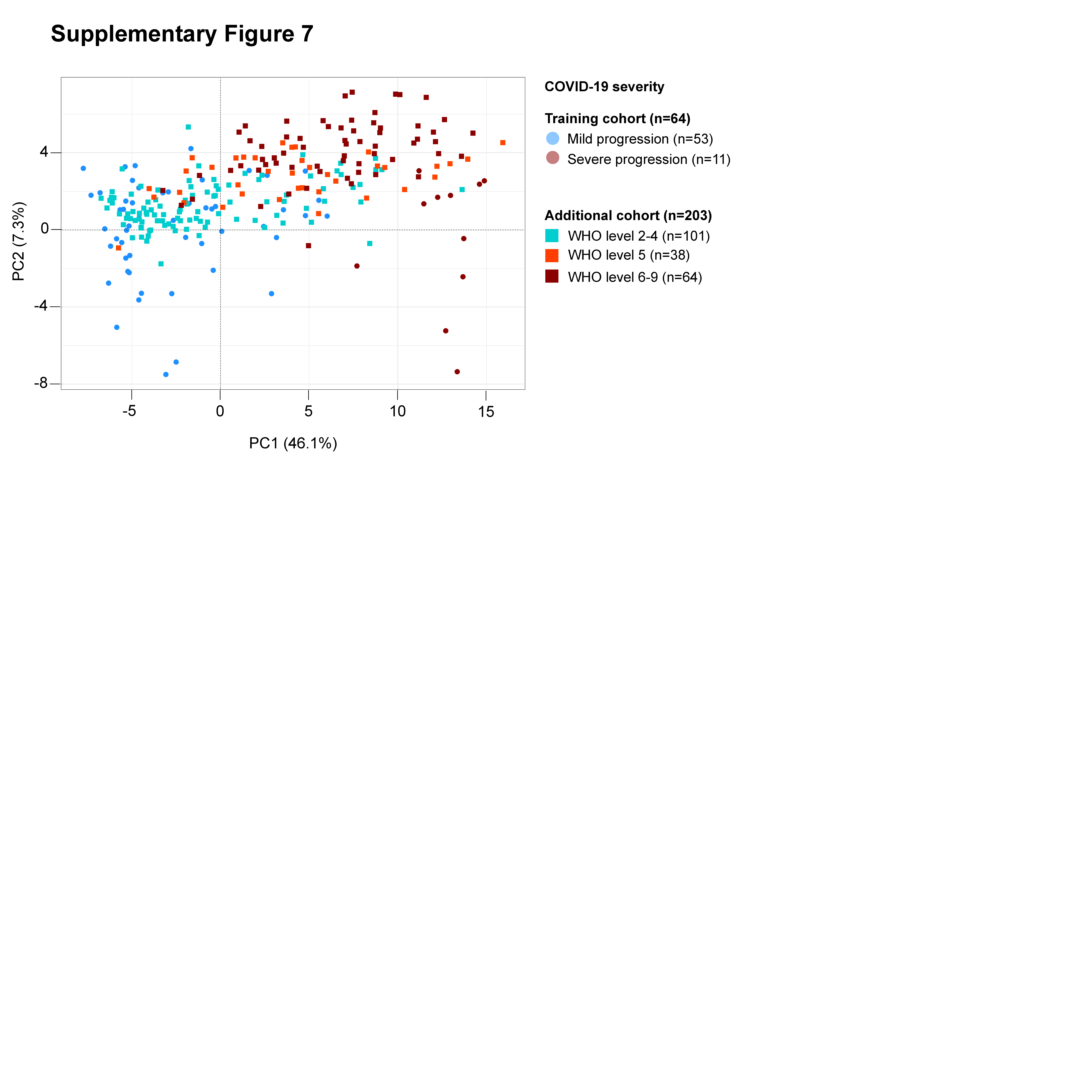

Supplement: Supplementary file 7 — Supplementary file7 (Supplementary Figure S7) (TIFF 1189 KB) PCA projection of 203 additional samples (bright squares), based on the 48-genes selection using the PCA weights established on the training cohort (faint circles) [file 10142_2024_1359_MOESM7_ESM.tiff]

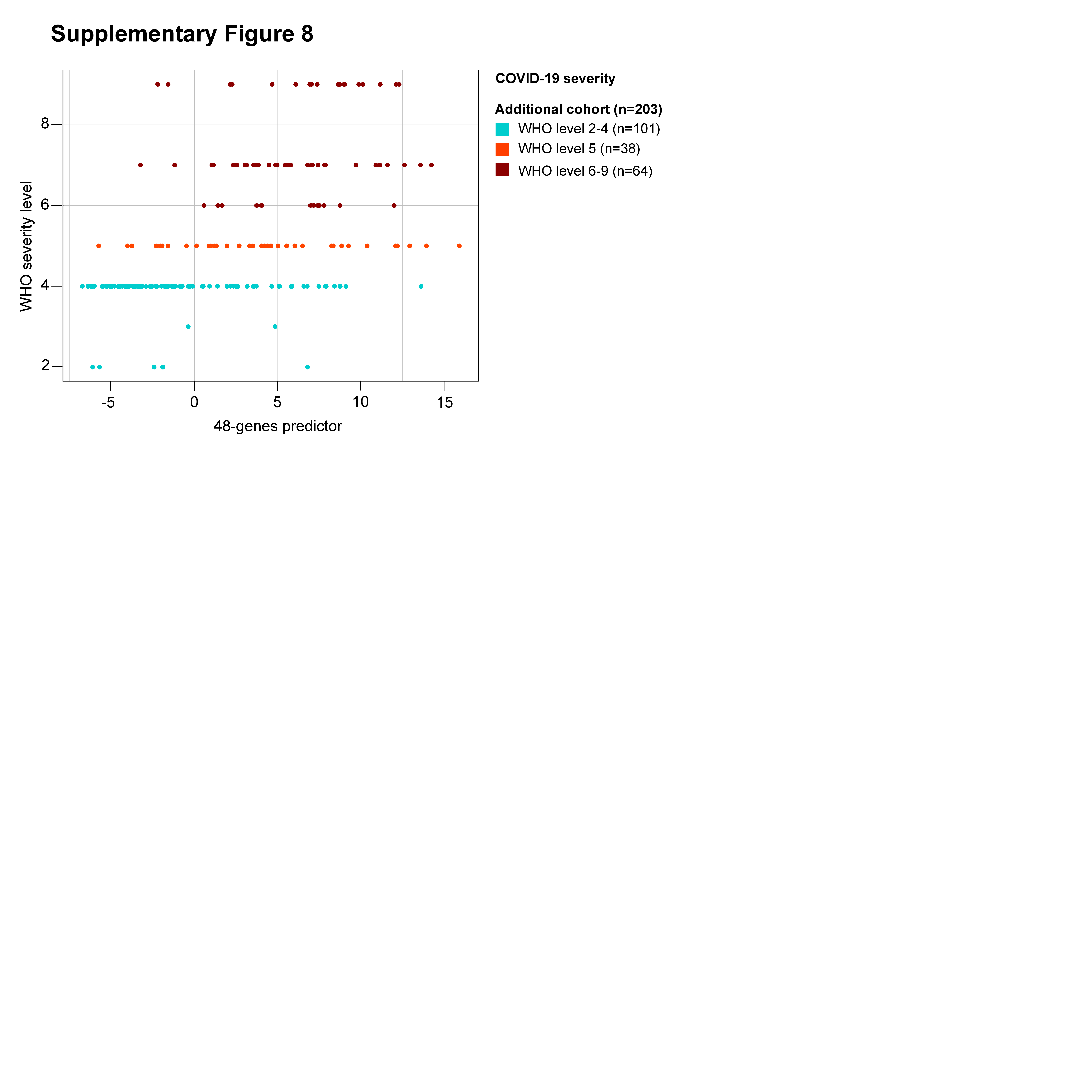

Supplement: Supplementary file 8 — Supplementary file8 (Supplementary Figure S8) (TIFF 867 KB) Correlation analysis between the 48-genes predictor and COVID-19 WHO severity levels in the Wang et al cohort24 [file 10142_2024_1359_MOESM8_ESM.tiff]
